# DDX11 loss causes replication stress and pharmacologically exploitable DNA repair defects

**DOI:** 10.1073/pnas.2024258118

**Published:** 2021-04-20

**Authors:** Nanda Kumar Jegadesan, Dana Branzei

**Affiliations:** ^a^The FIRC Institute of Molecular Oncology, Italian Foundation for Cancer Research, 20139, Milan, Italy;; ^b^Istituto di Genetica Molecolare, Consiglio Nazionale delle Ricerche, 27100, Pavia, Italy

**Keywords:** replication stress, homologous recombination, chemotherapy, BRCA1/2, DDX11

## Abstract

*DDX11* encodes an iron–sulfur cluster DNA helicase required for development, mutated, and overexpressed in cancers. Here, we show that loss of *DDX11* causes replication stress and sensitizes cancer cells to DNA damaging agents, including poly ADP ribose polymerase (PARP) inhibitors and platinum drugs. We find that DDX11 helicase activity prevents chemotherapy drug hypersensitivity and accumulation of DNA damage. Mechanistically, DDX11 acts downstream of 53BP1 to mediate homology-directed repair and RAD51 focus formation in manners nonredundant with BRCA1 and BRCA2. As a result, *DDX11* down-regulation aggravates the chemotherapeutic sensitivity of *BRCA1*/*2*-mutated cancers and resensitizes chemotherapy drug–resistant *BRCA1*/*2*-mutated cancer cells that regained homologous recombination proficiency. The results further indicate that DDX11 facilitates recombination repair by assisting double strand break resection and the loading of both RPA and RAD51 on single-stranded DNA substrates. We propose DDX11 as a potential target in cancers by creating pharmacologically exploitable DNA repair vulnerabilities.

## Significance

Replication stress can affect development and is a hallmark of cancers. Warsaw breakage syndrome is a developmental disorder caused by mutations in the conserved DDX11 DNA helicase. Here, using human cellular models of DDX11 deficiency, we report that DDX11 helicase prevents replication stress and mediates homology-directed repair via homologous recombination. Mechanistically, DDX11 promotes resection, enabling RPA and RAD51 focus formation, and acts nonredundantly with the RAD51 mediators BRCA1 and BRCA2. As a result, targeting DDX11 confers improved chemotherapy responsiveness in both chemotherapy-sensitive and drug-resistant BRCA1/2-mutated cancers that regained homologous recombination proficiency by suppressor mutation or somatic reversion. The results pinpoint DDX11 as a critical replication stress mitigating factor whose targeting can improve chemotherapeutic response in a range of cancers.

Faithful DNA replication and DNA repair processes are essential for genome integrity. Inherited mutations in *BRCA1* or *BRCA2* genes predispose to breast and ovarian cancer, among other types of malignancies such as pancreatic cancers and brain tumors (1). Mechanistically, BRCA1 and BRCA2 are critical for double strand break (DSB) repair by homologous recombination (HR) and for the protection of stalled replication forks by facilitating RAD51 filament formation (2).

Tumors with mutations in HR factors, the most widespread being those harboring mutations in *BRCA1* and *BRCA2*, are sensitive to chemotherapeutic drugs that block replication and cause DSBs (3). Platinum drugs, such as cisplatin, create intra- and interstrand adducts that require HR activities for DNA repair during replication and therefore are effective in killing HR-defective cancers. Analysis of the plateau of the survival curve of different cancers revealed that patients often develop resistance, and thus, alternative strategies are needed. The advent of PARP (poly ADP ribose polymerase) inhibitors (PARPi), including olaparib, which exhibit synthetic lethal effects when applied to cells and tumors defective in HR (4, 5), holds significant promise. PARP1, 2, and 3 are required to repair numerous DNA single-strand breaks (SSBs) resulting from oxidative damage and during base excision repair. When PARP enzymes are locally trapped at SSBs, they prevent fork progression and generate DSBs (6), which need to be repaired by BRCA1/2 and other HR factors (4, 5). While the synthetic lethality of PARPi and HR deficiency is being exploited clinically, many *BRCA*-mutated carcinomas acquire resistance to PARPi (2). Identifying key factors that are functionally linked with BRCA1/2 and/or PARP during replication stress response may indicate useful alternative or combinatorial chemotherapeutic strategies.

DDX11 is a conserved iron–sulfur (Fe–S) cluster 5′ to 3′ DNA helicase facilitating chromatin structure and DNA repair in manners that are not fully understood. Biallelic *DDX11* mutations in humans cause the developmental disorder Warsaw breakage syndrome (WBS), which presents overlaps with Fanconi anemia in terms of chromosomal instability induced by intra- and interstrand crosslinking (ICL) agents and with cohesinopathies in terms of sister chromatid cohesion defects (7, 8). DDX11 has also strong ties to cancer. Specifically, *DDX11* is highly up-regulated or amplified in diverse cancers, such as breast and ovarian cancers, including one-fifth of high-grade serous ovarian cancers (cBioPortal and The Cancer Genome Atlas [TCGA]). Moreover, DDX11 is required for the survival of advanced melanomas (9), lung adenocarcinomas (10), and hepatocellular carcinomas (11). In terms of molecular functions, DDX11 interacts physically with the replication fork component Timeless to assist replisome progression and to facilitate epigenetic stability at G-quadruplex (G4) structures and sister chromatid cohesion (12–16). Notably, DDX11 also contributes along 9–1-1, Fanconi anemia factors, and SMC5/6 to prevent cytotoxicity of PARPi and ICLs (17–20). However, if the DNA damage tolerance functions of DDX11 are relevant for tumorigenesis or cancer therapies remains currently unknown.

Here, we find that targeting *DDX11* sensitizes ovarian and other cancer cell lines to drug therapies involving cisplatin and the PARP inhibitor olaparib. We established *DDX11* knockout (KO) in HeLa uterine and U2OS osteosarcoma cancer cell lines and uncovered via chemical drug screens and immunofluorescence of DNA damage markers that they show typical hallmarks of increased replication stress. DDX11 helicase activity and the Fe–S domain are critical to prevent cellular sensitization to olaparib and ICLs and to avert accumulation of DSB markers. Mechanistically, we uncover that DDX11 facilitates homology-directed repair of DSBs and RAD51 focus formation downstream of 53BP1. Importantly, DDX11 is required for viability in *BRCA1*-depleted cells that are resistant to chemotherapy by concomitant depletion of *53BP1*, *REV7*, and other shieldin components (21, 22), indicating roles for DDX11 in the activated BRCA2-dependent HR pathway, often accounting for the resistance of *BRCA1*-mutated tumors (2). DDX11 DNA repair function is nonredundant with BRCA1 and BRCA2 pathways, facilitating resection and loading of both RPA and RAD51 on single-stranded DNA substrates. Altogether, our results define a DDX11-mediated DNA repair pathway that creates pharmaceutically targetable vulnerabilities in cancers.

## Results

### DDX11 Loss Sensitizes Ovarian Cancers to PARPi and Cisplatin.

**Fig. 1.**
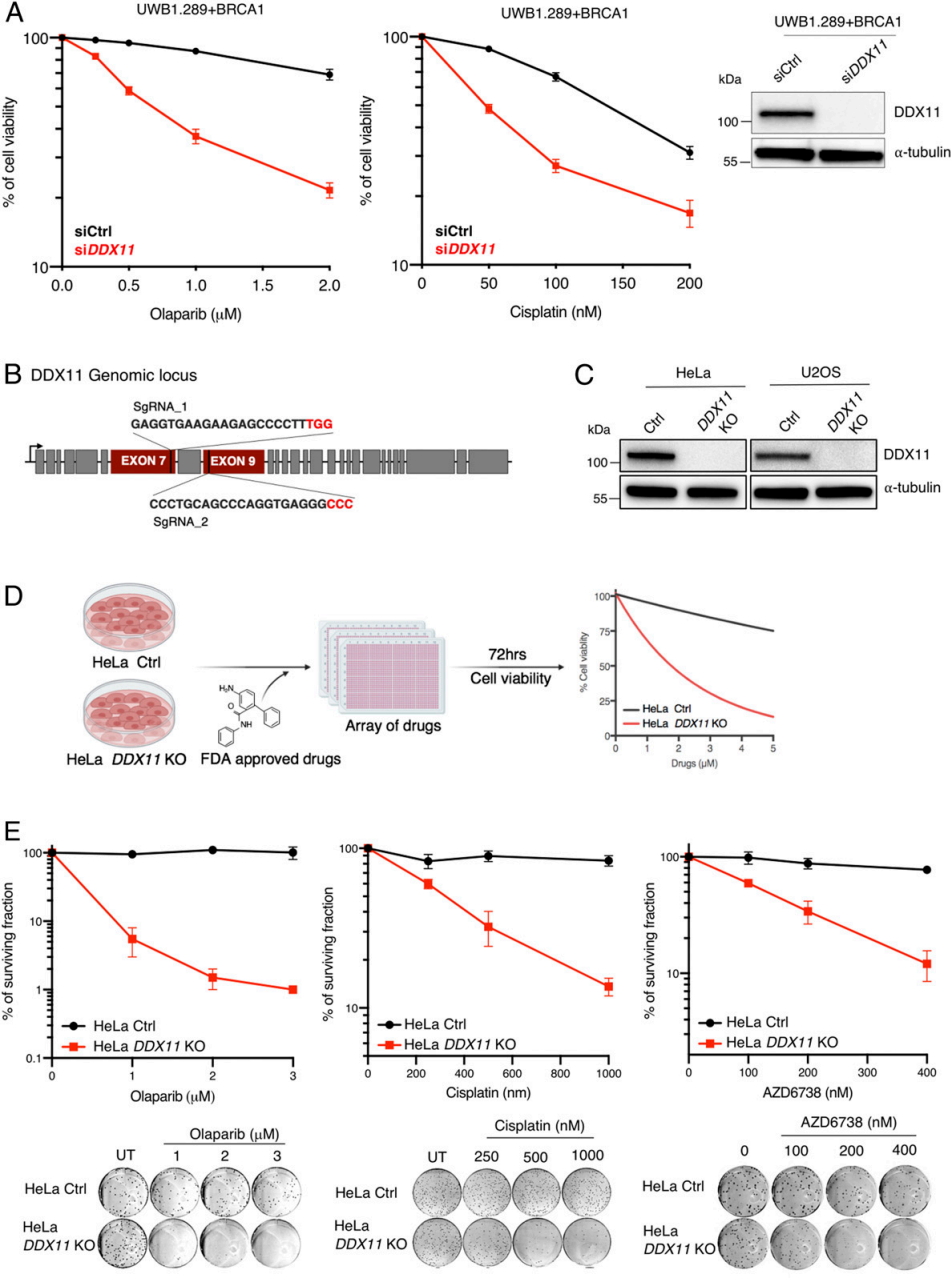
Establishment of *DDX11* KO in cancer cell lines and identification of synthetic lethal drugs (*A*) Cell viability assay of ovarian cancer cell line UWB1.289 + BRCA1 transfected with siCtrl and si*DDX11*. Cells were treated with olaparib and cisplatin with the indicated drug concentrations (*n* = 3). (*Right*) Corresponding Western blot. Error bars show average ± SEM. (*B*) Schematic representation of *DDX11* genomic locus, targeted by the CRISPR-paired guide RNAs at exons 7 and 9 to establish KO in HeLa and U2OS cell lines. (*C*) Western blot analysis of DDX11 in HeLa and U2OS cell lines to assess *DDX11* targeting with CRISPR-paired guide RNAs. (*D*) Schematic representation of synthetic lethal drug screen (Food and Drug Administration [FDA]–approved drugs) in HeLa Ctrl and *DDX11* KO cells in which cell viability was determined 72 h after drug treatment with 64 FDA-approved drugs at different concentrations using CellTiter-Glo (*n* = 2). (*E*) Colony formation assay of HeLa Ctrl and *DDX11* KO cells exposed to olaparib (*n* = 2), cisplatin (*n* = 3), and ATRi AZD6738 (*n* = 3) with the indicated drug concentrations. Colonies were stained with crystal violet after 10 to 15 d of incubation. For cisplatin, after 1 h of acute treatment, cells were allowed to grow in normal media. Error bars show average ± SEM.

*DDX11* is overexpressed in various cancers and amplified in 21% of ovarian serous cystadenocarcinoma (TCGA Pan-Cancer Atlas and CBioPortal). Moreover, Kaplan–Meier analysis of the probability of survival of cancer patients divided in two groups by *DDX11* median expression shows that high levels of *DDX11* expression significantly correlate with decreased overall survival of patients with ovarian and lung cancers (*SI Appendix*, Fig. S1*A*). We aimed to examine whether targeting *DDX11* by small interfering RNA (siRNA) affects cell viability in ovarian cancer cell lines exposed to baseline therapy constituted by cisplatin and olaparib, as previous results suggested a role for vertebrate DDX11 in the tolerance of such lesions (17, 18, 23). Silencing of *DDX11* using siRNA reduced cell viability in a series of ovarian cancer cell lines, namely UWB1.289 + BRCA1 (Fig. 1*A*), OVCAR8, IGROV1, and COV362 (*SI Appendix*, Fig. S2*A*) as assessed by crystal violet staining of viable cells upon chronic cisplatin and olaparib drug treatment for 5 to 6 d (Fig. 1*A* and *SI Appendix*, Fig. S2*A*). Thus, *DDX11* targeting sensitizes ovarian cancer cell lines to chemotherapy.

### Establishment of *DDX11* KO and Identification of Synthetic Lethal Drugs.

The molecular mechanism of DDX11 in DNA damage resistance of cancer cell lines is unclear. We established *DDX11* KO in HeLa and U2OS cell lines using CRISPR-paired guide RNAs targeting exons 7 and 9 (Fig. 1*B*). We confirmed *DDX11* KOs of selected clones using Western blot analysis (Fig. 1*C*) and Sanger sequencing to identify the indels at the *DDX11* genomic locus caused by the Cas9 nuclease (*SI Appendix*, Fig. S2*B*). The absence of DDX11 from selected clones was also confirmed by the lack of immunofluorescence staining of DDX11 in HeLa and U2OS (*SI Appendix*, Fig. S2*C*). *DDX11* KO showed no proliferation defects in HeLa but caused a proliferation delay in U2OS cells (*SI Appendix*, Fig. S2*D*). These differences in proliferation associated with *DDX11* KO may relate to the p53 status (8, 15), although other explanations are possible.

To investigate DDX11 functions in chemotherapy sensitization of cancer cells, we performed synthetic lethality drug screens in HeLa control (Ctrl) and *DDX11* KO cells using 64 US Food and Drug Administration–approved drugs affecting different pathways (*SI Appendix*, Table S1). After 72 h of posttreatment at six various concentrations for each drug, cell viability was determined using CellTiter-Glo (Fig. 1*D*, Dataset S1, and *SI Appendix*, Table S2). We found that HeLa *DDX11* KO cells are sensitized by various drugs that include ATR inhibitors (ATRi), Bleomycin, Mitoxanthrone and other topoisomerase II poisons, PARPi, and platinum drugs (*SI Appendix*, Tables S1 and S2 and Dataset S1), several of which cause DSBs. Among top hits, we validated olaparib and mitomycin C using cell viability assays (*SI Appendix*, Fig. S3*A*). Moreover, using a colony formation assay, we validated *DDX11* KO hypersensitivity toward olaparib, cisplatin, and the ATRi AZD6738 (Fig. 1*E*) and VE-821 (*SI Appendix*, Fig. S3*B*). Notably, knockdown of *DDX11* in several nonmalignant cell lines, namely in hTERT RPE-1 (retinal epithelial), MCF10A (breast), and BJ (fibroblasts), caused only minor effects and at higher drug concentrations than those affecting cancer cell lines (*SI Appendix*, Fig. S3*C*).

### DDX11 Loss Causes Persistent DNA Damage Accumulation.

**Fig. 2.**
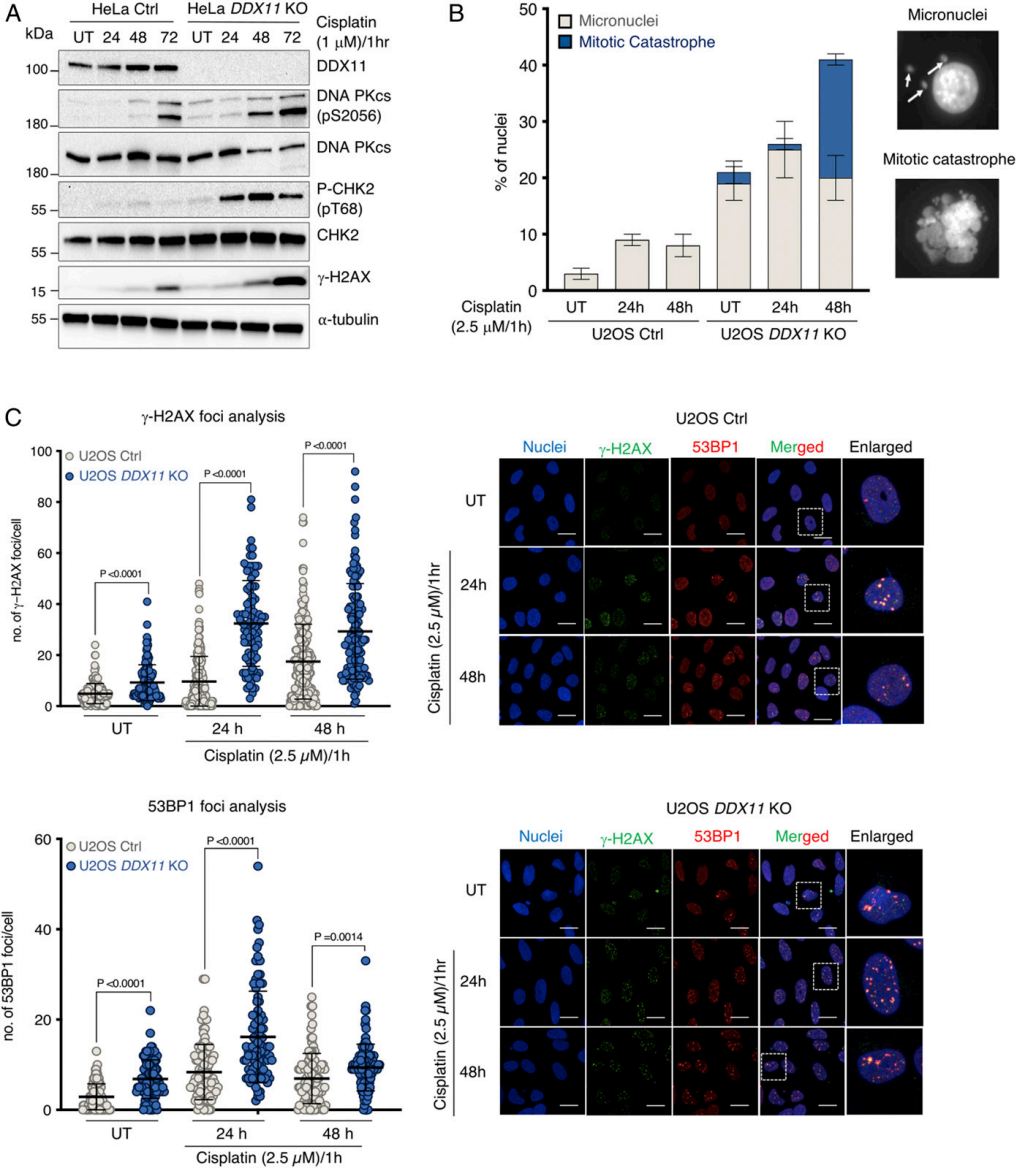
DDX11 loss associates with persistent DNA damage accumulation and micronucleation. (*A*) HeLa Ctrl and *DDX11* KO cells were treated with cisplatin (1 μM) for 1 h and allowed to recover for 72 h, during which DNA damage markers were analyzed by Western blotting at the indicated time points (*n* = 2). (*B*) Quantification of micronuclei and mitotic catastrophes in U2OS Ctrl and *DDX11* KO cells in untreated conditions and upon recovery from an acute cisplatin treatment (2.5 μM for 1 h). Error bars show average ± SEM. (*C*) Quantification and representative micrographs of γ-H2AX and 53BP1 foci in U2OS Ctrl and *DDX11* KO cells recovering from an acute treatment with cisplatin (2.5 μM for 1 h). (Scale bar, 10 μm.) *n* = 2. Statistical analysis of foci was performed using Student’s *t* test. Error bars show average ± SD.

We next examined whether the sensitivity of HeLa *DDX11* KO cells to chemotherapeutic drugs associates with the accumulation of DNA damage. The recovery from acute cisplatin treatment in HeLa *DDX11* KO cells resulted in increased γ−H2AX and phosphorylated CHK2 (CHK2-P), indicative of persistent DSBs (Fig. 2*A*). Moreover, *DDX11* KO cells showed increased activation of DNA-dependent protein kinase, catalytic subunit (PKcs) involved in nonhomologous end joining (NHEJ) (24) as detected by its autophosphorylation (pS2056) (Fig. 2*A*). We further observed an increase in micronuclei and mitotic catastrophes after the recovery from acute cisplatin treatment or from olaparib treatment in both HeLa and U2OS cells knocked out for *DDX11* in comparison with control cell lines under the same experimental conditions (Fig. 2*B* and *SI Appendix*, Fig. S4*A*). In the same vein, γ−H2AX and 53BP1 foci were significantly increased in U2OS *DDX11* KO cells in unperturbed conditions and upon recovery from cisplatin and olaparib drug treatments (Fig. 2*C* and *SI Appendix*, Fig. S4*B*). Altogether, the results indicate that the loss of DDX11 compromises genomic stability and leads to accumulation of DNA damage.

### DDX11 Helicase Activity Averts DNA Damage Accumulation and Chemotherapy Sensitivity.

**Fig. 3.**
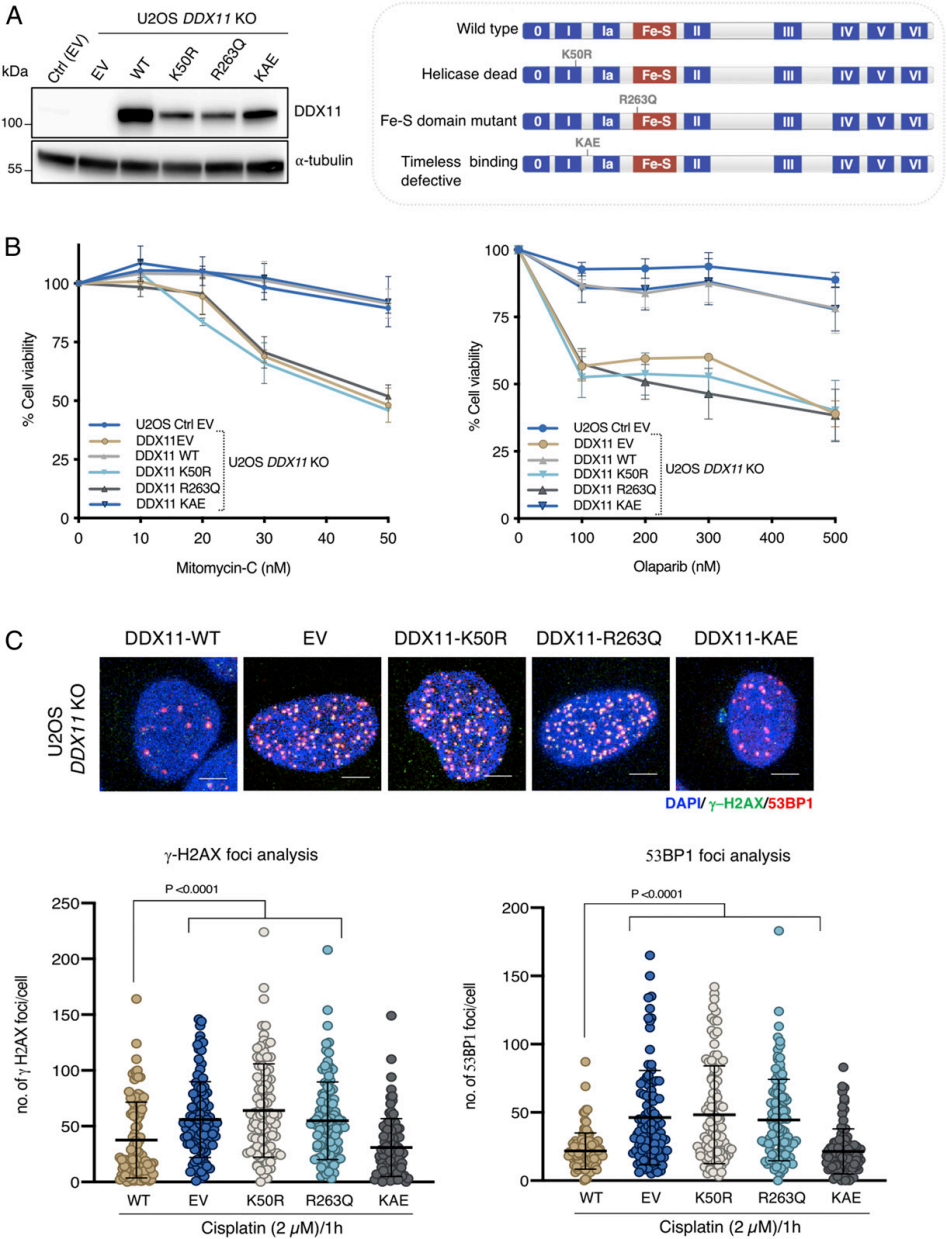
DDX11 helicase activity averts DNA damage accumulation and damage sensitivity. (*A*) Western blot analysis of U2OS Ctrl and *DDX11* KO cells complemented with EV, WT DDX11, DDX11 helicase dead (K50R), mutation in Iron–Sulfur cluster domain (R263Q), and DDX11-Timeless interaction defective motif (KAE). (*Right*) Schematic representation of *DDX11* and its associated mutations. (*B*) Cell viability of U2OS Ctrl and *DDX11* KO cells complemented with EV and DDX11 variants upon mitomycin C and olaparib drug treatment. Cell viability was measured using crystal violet after 5 d of incubation in the presence of the drugs at the indicated concentrations (*n* = 3). Error bar shows average ± SEM. (*C*) Quantification and representative micrographs of 53BP1 and γ-H2AX foci in U2OS Ctrl and *DDX11* KO cells complemented with different DDX11 variants after 24 h of recovering from 1 h treatment with cisplatin (2 μM). (Scale bar, 10 μm.) *n* = 2. Statistical analysis was performed using Student’s *t* test. Error bar shows average ± SD.

DDX11 helicase activity along its Fe–S domain are essential for unwinding DNA replication forks (12, 25, 26). Moreover, the interaction between DDX11 and Timeless facilitates normal replication fork speed and the processing of G4 secondary structures (8, 13, 16). To inquire on the activities/interactions of DDX11 facilitating cell survival in response to chemotherapy-induced lesions, we stably complemented U2OS *DDX11* KO by ectopically overexpressing different DDX11 variants, namely DDX11-WT (wild type), DDX11-K50R (helicase dead), DDX11-R263Q (Fe–S domain mutated), and DDX11-KAE (lacking interaction with the Timeless-Tipin complex) (Fig. 3*A*). We found that the helicase activity and Fe–S domain are critical for cellular viability upon olaparib and mitomycin C drug treatment, as these mutants showed similar sensitivity levels to U2OS *DDX11* KO cells carrying EV (empty vector) (Fig. 3*B*). Of interest, the Timeless interaction was not essential in this process (Fig. 3*B*). In the same line with the cellular viability results, we found that K50R and R263Q DDX11 variants are severely defective in preventing γ−H2AX and 53BP1 DNA damage foci accumulation upon recovery from cisplatin treatment, whereas the KAE mutant had only minor effects in this regard (Fig. 3*C*). Thus, DDX11 helicase is critical to prevent DNA damage accumulation and chemotherapy sensitivity.

### DDX11 Promotes Homology-Directed Repair of DSBs and RAD51 Foci Formation Nonredundantly with BRCA1 and BRCA2.

**Fig. 4.**
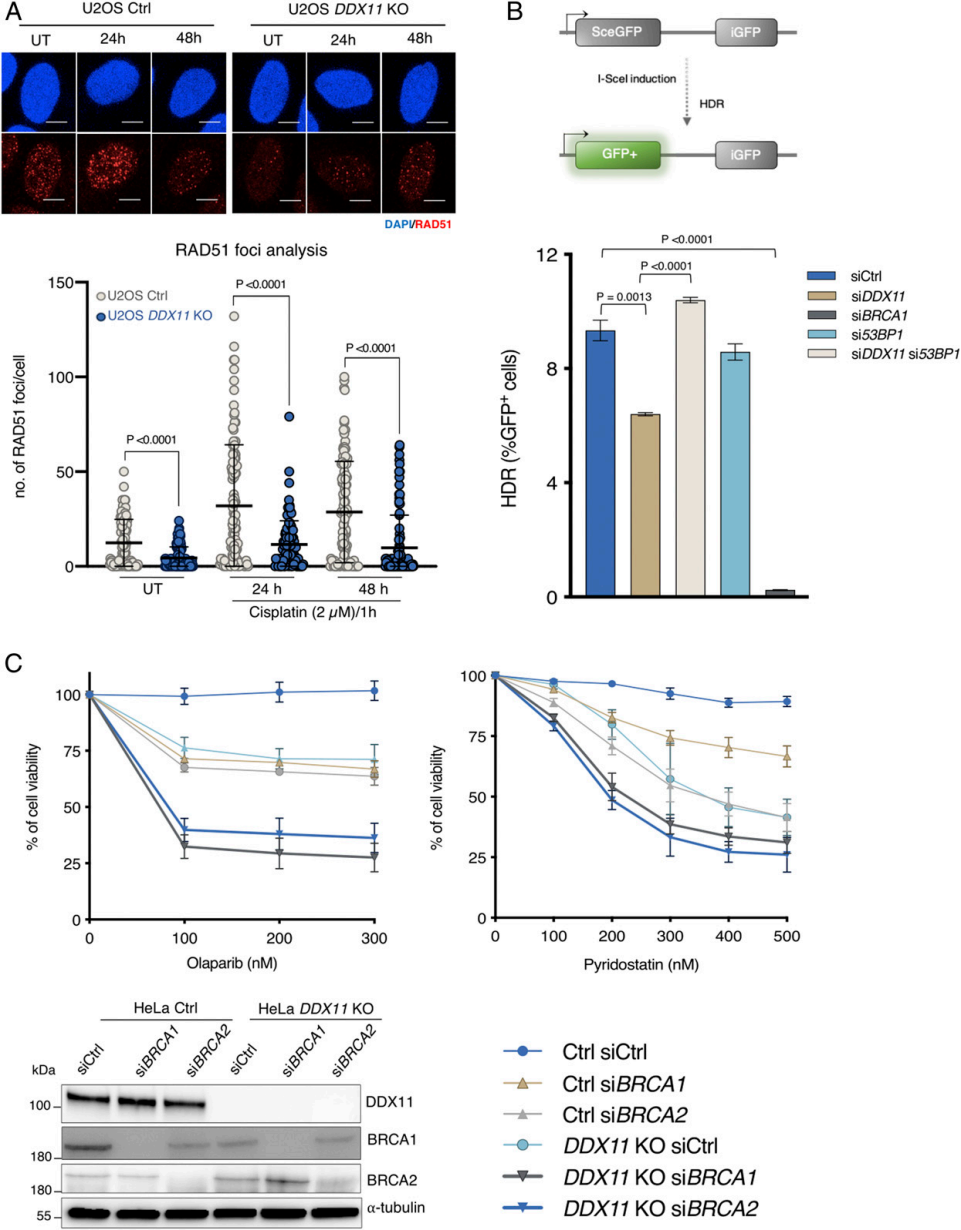
DDX11 promotes homology-directed repair of DSBs and RAD51 foci formation nonredundantly with BRCA1 and BRCA2. (*A*) Quantification and representative micrographs of RAD51 focus formation in U2OS Ctrl and *DDX11* KO recovering from 1 h treatment with cisplatin (2.5 μM). (Scale bar, 10 μΜ.) *n* = 2. Statistical analysis was performed using Student’s *t* test. Error bar shows average ± SD. (*B*) U2OS TRI DR-GFP cells were transfected with indicated siRNAs, and Sce-I was induced by adding doxycycline after 48 h of siRNA transfection. Fluorescence-activated cell sorting analysis was performed after 72 h of doxycycline induction (*n* = 3). Schematic representation of the assay is shown above. Error bars show average ± SEM. (*C*) Cell viability assay of HeLa Ctrl and *DDX11* KO cells transfected with siCtrl, si*BRCA1*, and si*BRCA2*. Cells were treated with olaparib and Pyridostatin with the indicated drug concentrations for 72 h, and cell viability was measured using crystal violet staining (*n* = 3). (*Bottom Left*) Corresponding Western blot is shown. Statistical analysis was performed using Student’s *t* test. Error bars show average ± SEM.

Cells defective in HR often show sensitivity to olaparib and cisplatin drugs. Studies in chicken DT40 cells proposed a role for DDX11 in sister chromatid exchanges and RAD51 focus formation upon replication damage (17). However, no defect in RAD51 focus accrual was observed in DDX11-deficient lymphoblasts derived from a WBS patient (18). To address whether the accumulation of DNA damage and hypersensitivity in *DDX11* KO cells may stem from a defect in HR, we analyzed RAD51 foci in U2OS Ctrl and *DDX11* KO cells in unperturbed conditions and upon recovery from cisplatin treatment. We found a significant decrease in RAD51 foci in *DDX11* KO cells in both experimental conditions (Fig. 4*A*). To address whether the observed decrease in RAD51 foci in *DDX11* KO cells exposed to DNA damage relates to a role for DDX11 in DSB repair by homology-directed repair, we investigated the efficiency of the latter using a direct repeats GFP (DR-GFP) assay (27). We found that si*DDX11* cells had significantly lower homology-directed repair efficiency compared with control cells but were not as drastically defective as si*BRCA1* cells (Fig. 4*B* and *SI Appendix*, Fig. S5*A*). The loss of 53BP1 can enhance HR repair by allowing resection (28, 29). Notably, we found that si*53BP1* rescued the homology-directed repair defect of si*DDX11* cells in the DR-GFP assay (Fig. 4*B* and *SI Appendix*, Fig. S5*A*). Colony formation and cell viability assays further revealed that the loss of 53BP1 in *DDX11* KO cells partly rescued their cisplatin and olaparib sensitivity (*SI Appendix*, Fig. S5*B* and see below). Because BRCA1 and BRCA2 are critical RAD51 mediators, we analyzed the functional interaction with DDX11. We found that double mutants between *DDX11* KO and either si*BRCA1* or si*BRCA2* are more sensitive than single mutants toward olaparib, cisplatin, and G4 stabilizing drugs, such as Pyridostatin and Telomestatin, shown previously to sensitize DDX11 and BRCA1/2 mutant cells (8, 30) (Fig. 4*C* and *SI Appendix*, Fig. S6). Thus, DDX11 participates in the homology-directed repair of DSBs and RAD51 focus formation but acts nonredundantly with BRCA1 and BRCA2 in DNA repair.

### DDX11 Loss Resensitizes *BRCA1*-Deficient Cells with Acquired Drug Resistance.

**Fig. 5.**
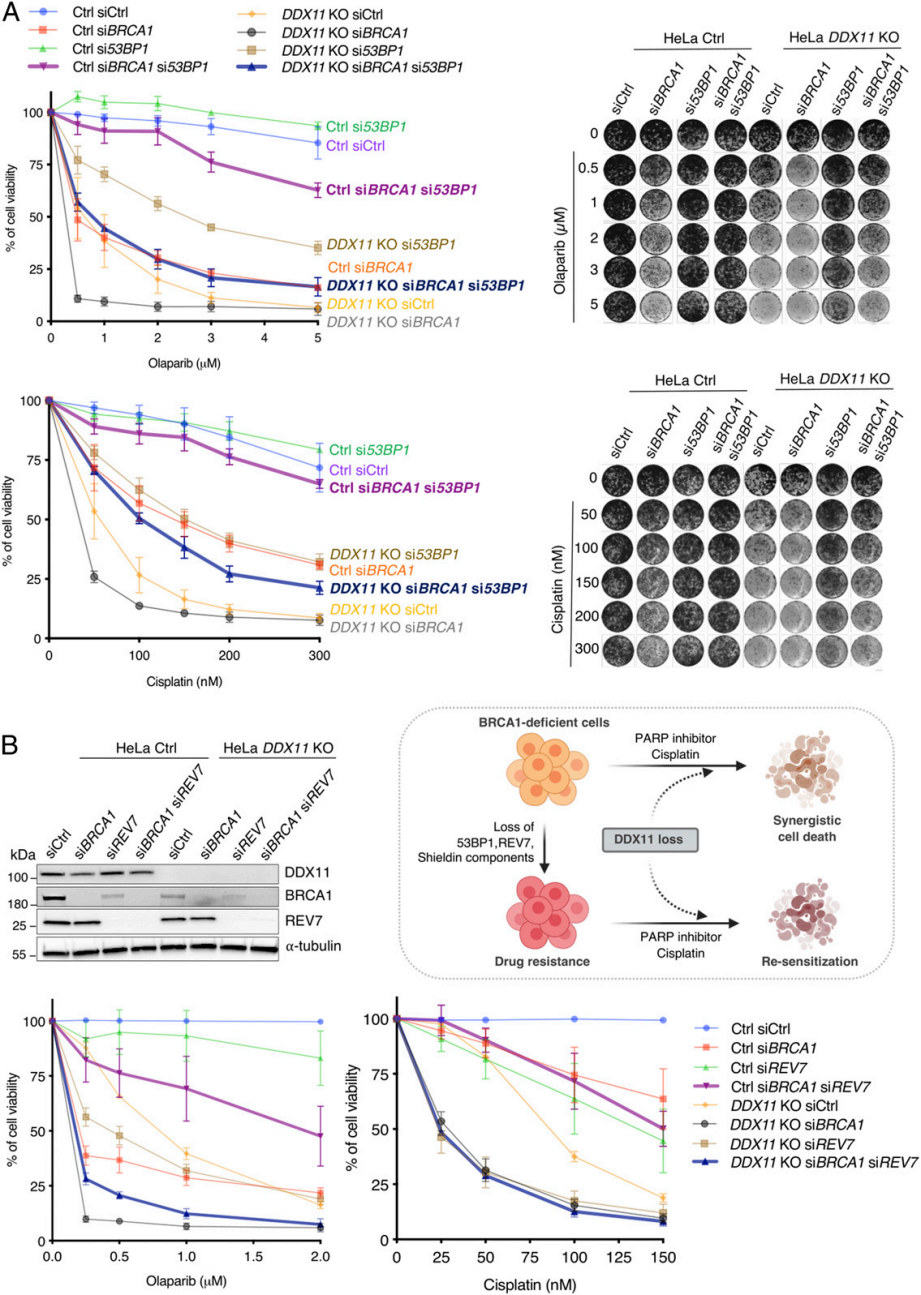
DDX11 loss resensitizes si*BRCA1* cells that acquired drug resistance via 53BP1 or shieldin loss of function. (*A*) Cell viability assay of HeLa Ctrl and *DDX11* KO cells transfected with indicated siRNAs. Cells were treated with olaparib and cisplatin with the indicated drug concentrations for 5 to 6 d (*n* = 3). Cell viability was determined by using crystal violet staining. (*Right*) Corresponding plates are shown. Its corresponding Western blot is shown in *SI Appendix*, Fig. S7. Error bars show average ± SEM. (*B*) Cell viability assay of HeLa Ctrl and *DDX11* KO cells transfected with indicated siRNAs. Cells were treated with olaparib and cisplatin with the indicated drug concentrations for 5 to 6 d (*n* = 3). Cell viability was determined by using crystal violet staining. (*Top Left*) Corresponding Western blot is shown. Error bars show average ± SEM. (*Top Right*) Model for DDX11 loss in BRCA1 mutant cancer cells are synergistic, and resensitization of BRCA1 drug-resistant cancer cells to chemotherapeutic drugs.

Previous studies highlighted that the loss of 53BP1 in *BRCA1* mutated/null tumors leads to drug resistance (31). Because si*53BP1* caused higher improvement in the viability of si*BRCA1* cells compared with *DDX11* KO treated with DNA damage (*SI Appendix*, Fig. S7), we asked whether DDX11 is required for the HR pathway activated in si*BRCA1* si*53BP1* cells. Strikingly, the loss of DDX11 resensitized si*53BP1* si*BRCA1* cells toward both olaparib and cisplatin (Fig. 5*A*). Moreover, we assessed whether DDX11 is required for the viability of BRCA1-depleted cells rendered resistant to chemotherapeutic drugs via removal of other shieldin components (reviewed in ref. 21). Depletion of *REV7* and shieldin components *FAM35A* and *C20orf196* rendered *BRCA1*-depleted cells resistant to olaparib and cisplatin but did not rescue the sensitivity of *DDX11* KO cells (Fig. 5*B* and *SI Appendix*, Fig. S8). Importantly, the viability of BRCA1-depleted cells rendered resistant by mutations in REV7 and shieldin largely depended on DDX11 (Fig. 5*B* and *SI Appendix*, Fig. S8). Thus, DDX11 is required for viability in response to chemotherapy in *BRCA1*-depleted cells that acquire resistance via inactivation of 53BP1, REV7, and shieldin components.

### DDX11 Is Complementary with BRCA2 in Facilitating DNA Repair and Genome Stability.

**Fig. 6.**
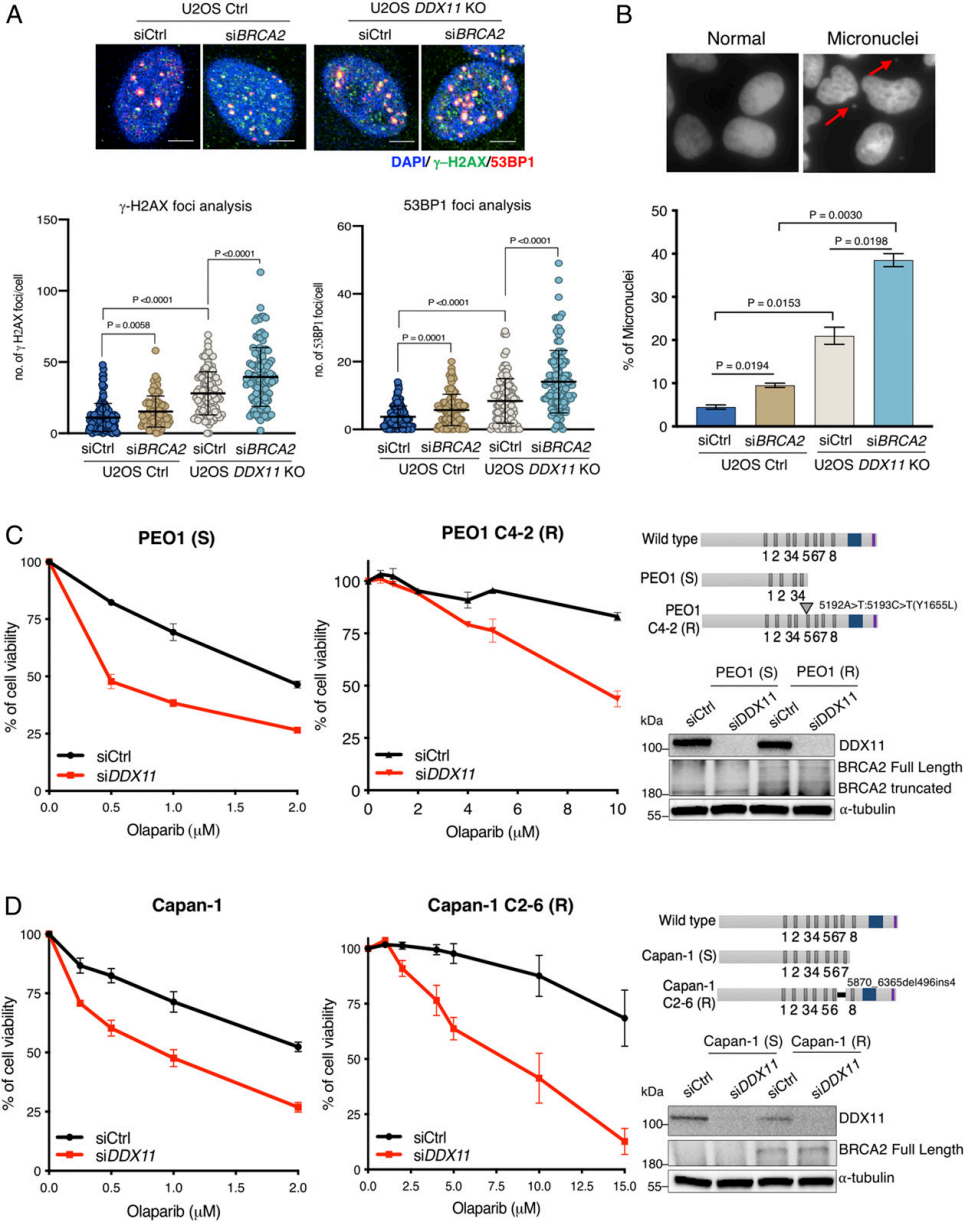
DDX11 is complementary with BRCA2 in facilitating DNA repair and genome stability (*A*) Quantification and representative micrographs of γ-H2AX and 53BP1 foci in U2OS Ctrl and *DDX11* KO cells after 72 h of siCtrl and si*BRCA2* transfection. (Scale bar, 10 μm.) *n* = 2. Statistical analysis was performed using Student’s *t* test. Error bars show average ± SD. (*B*) Quantification and representative micrographs of micronuclei in U2OS Ctrl and *DDX11* KO cells after 72 h of indicated siRNAs transfection, *n* = 2. Statistical analysis was performed using Student’s *t* test. Error bars show average ± SEM. (*C*) Cell viability assay of PEO1 (S) and C4-2 (R) cells transfected with siCtrl and si*DDX11*. Cells were treated with olaparib with the indicated drug concentrations for 5 to 6 d (*n* = 3). Cell viability was determined using crystal violet staining. (*Top Right*) Schematic representations of BRCA2 mutations and (*Bottom Right*) Western blot of DDX11 and BRCA2 variants. Error bars show average ± SEM. (*D*) Cell viability assay of Capan-1 (S) and Capan-1 (R) cells transfected with indicated siRNAs. Cells were treated with olaparib with the indicated drug concentrations for 5 to 6 d (*n* = 3). Cell viability was determined using crystal violet staining. (*Top Right*) Schematic representations of BRCA2 mutations and (*Bottom Right*) Western blot of DDX11 and BRCA2 variants. Error bars show average ± SEM.

We further analyzed the functional interaction between DDX11 and BRCA2, as BRCA2 is critical for DNA repair in si*BRCA1* si*53BP1* cells (28, 32), whose viability also depends on DDX11 (Fig. 5*A*). We found that *DDX11* KO increases spontaneous DNA damage in BRCA2-depleted U2OS cells as observed by increased 53BP1 and γ−H2AX DNA damage foci (Fig. 6*A*) and higher levels of micronucleation (Fig. 6*B*) in double mutants compared with the single inactivation of *DDX11* and *BRCA2*. To analyze the functional interaction between DDX11 and BRCA2 in tumor cells upon chemotherapy drug treatment, we examined the effect of si*DDX11* in ovarian and pancreatic tumors that carry BRCA2 mutations (PEO1 and Capan-1) and are sensitive to chemotherapy as well as the corresponding tumor cell lines that acquired PARPi resistance by secondary mutations, resulting in restoration of *BRCA2* (PEO1 C4-2 and Capan-1 C2-6) (33, 34). Notably, knockdown of *DDX11* in PEO1 and Capan-1 sensitive (S) as well as resistant (R) clones rendered the BRCA2-mutated tumor cells sensitive to olaparib (Fig. 6 *C* and *D*). Thus, DDX11 loss sensitizes both HR-deficient and HR-proficient cancer cells to agents that necessitate repair via HR.

### DDX11 Facilitates DSB Resection and RPA Loading.

**Fig. 7.**
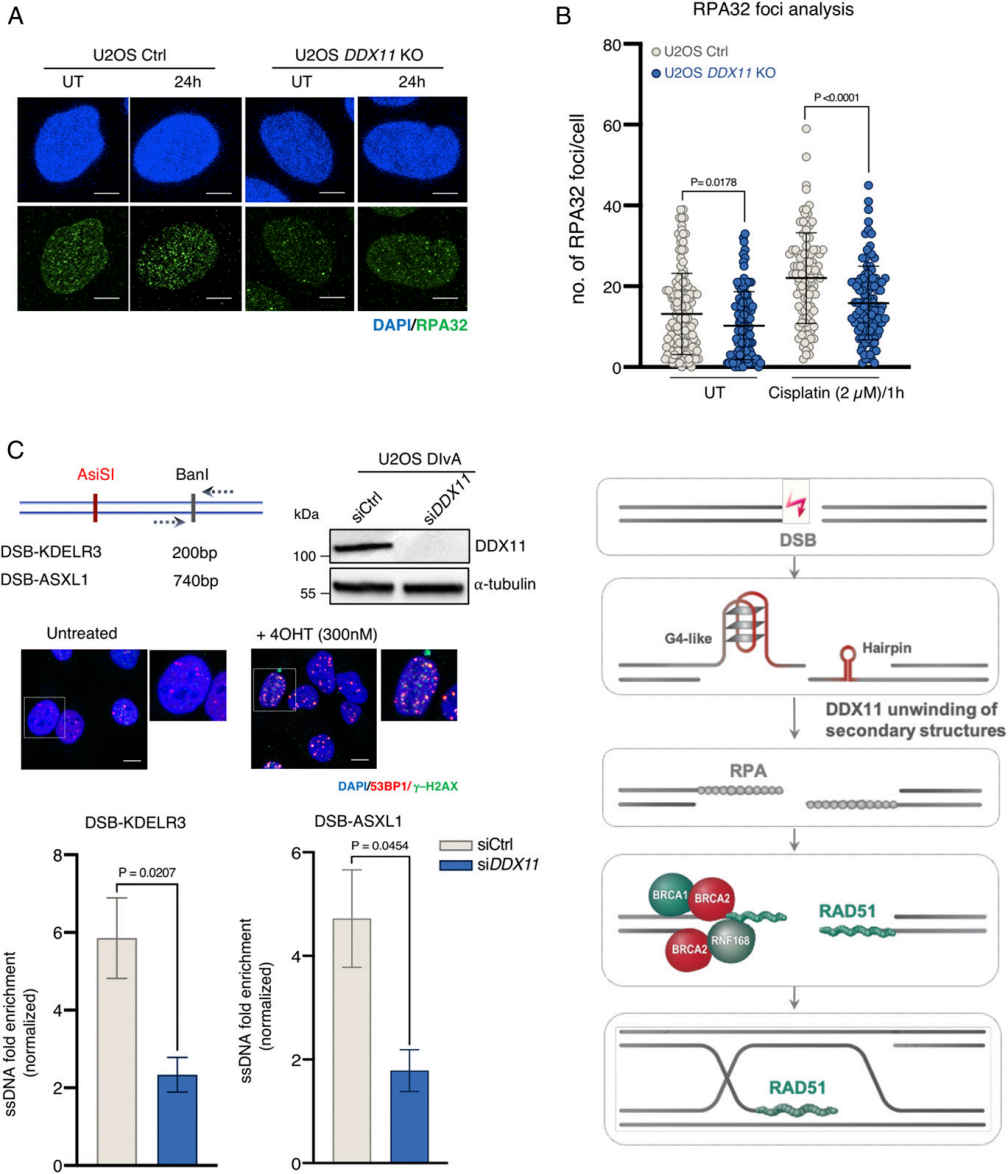
DDX11 facilitates resection and RPA loading to ssDNA substrates. (*A*) Quantification and representative micrographs of RPA32 foci in U2OS Ctrl and *DDX11* KO cells recovering from an acute treatment with cisplatin (2.5 μM for 1 h) after 24 h. (Scale bar, 10 μm.) *n* = 2. Statistical analysis of foci was performed using Student’s *t* test. Error bars show average ± SD. (*B*) U2OS DIvA cells were transfected with siCtrl and si*DDX11* followed by the addition of 4OHT (300 nM) to induce AsiSI-mediated DSBs after 48 h of post-transfection. Resection assay at the two different regions (KDELR3 and ASXL1) were analyzed after 4 h of 4OHT by real-time PCR. Values were normalized against the amount of ssDNA detected in control cells prior to 4OHT treatment, *n* ≥ 3. Corresponding Western blot and immunofluorescence for AsiSI-induced DSBs is shown. (Scale bar, 10 μm.) Statistical analysis was performed using Student’s *t* test. Error bars show average ± SEM. (*C*) Model for the DDX11 proposed role in resolving secondary structures upon DSB end resection to facilitate RPA loading and subsequently RAD51 nucleofilament formation required for homologous recombination-mediated DSB repair (see text for details).

*DDX11* KO cells show a BRCAness state in terms of chemotherapy sensitivity and RAD51 focus formation upon DNA damage. To understand whether DDX11 affects a step related to RAD51 loading or filament extension/stability or may influence steps upstream of RAD51 nucleation, we examined RPA32 focus formation. We found a significant decrease in RPA32 foci in U2OS *DDX11* KO cells in both unperturbed conditions and upon recovery from cisplatin drug treatment (Fig. 7*A*). To inquire if DDX11 may facilitate the levels of single-stranded DNA (ssDNA) substrates to which RPA is loaded, we used the DSB inducible via the AsiSI (DIvA) U2OS cell line, which allows to induce clean DSBs throughout the genome (35). In this cell line, 4-hydroxytamoxifen (4OHT) treatment induces the relocalization of a stably expressed restriction enzyme (AsiSI) that triggers the production of multiple DSBs at annotated positions across the genome and increased 53BP1 and γ−H2AX foci (Fig. 7*B*). We quantitatively measured the formation of ssDNA upon DSB induction by AsiSI using quantitative PCR at two different DSB break sites, KDELR3 (∼200 base pairs [bp]) and ASXL1 (∼740 bp), downstream of the AsiSI-induced break site (35), observing a significant decrease in ssDNA formation in si*DDX11* U2OS DIvA cells at both loci (Fig. 7*B*). Altogether, these results reveal that DDX11 promotes the formation of a ssDNA substrate suitable for RPA loading and subsequently for RAD51 nucleofilament formation and HR-mediated repair (Fig. 7*C*).

## Discussion

The genome of cancer cells is highly unstable and acquires resistance to the backbone therapies, which suggests that alternative approaches are needed. Moreover, development of new biomarker assays beyond *BRCA* mutations of chemotherapy responsiveness would facilitate efforts to optimize current therapies (36). The present work meets both needs. We uncover DDX11 as a potential target in tumors, including *BRCA1*/*2*-mutated cancers that acquired chemotherapeutic resistance, and pinpoint DDX11 mutations/loss as a useful biomarker of responsiveness to platinum drugs, PARP inhibitors, and ATRi.

Successful chemotherapies exploit fundamental vulnerabilities of cancer cells to increase replication stress, causing specific cell death. Here, we find that targeting *DDX11*, a gene whose up-regulation in several cancers correlates with poor patient prognosis (9–11), sensitizes ovarian, uterine, and other cancers to PARP inhibitors and platinum chemotherapies by causing an accumulation of DNA damage and mitotic instability. These phenotypes could result from DDX11 roles in repairing replication-associated lesions and/or preventing their formation. Here, we provide evidence that DDX11 facilitates homology-directed DNA repair of DSBs and potentially of other replication-associated lesions by promoting RPA loading on ssDNA substrates and subsequently RAD51 focus formation. This role of DDX11 in mitigating replication stress relies on its helicase activity and is especially relevant in replication stress conditions induced by DNA damaging drugs, correlating with the ability of cancer cells to tolerate the chemotherapy-induced DNA damage.

Once DSBs are formed, their repair choice is being directed by 53BP1 binding to the break ends, which favors NHEJ-mediated repair while preventing resection (22, 28, 32). End resection, favored by BRCA1 engagement of the DSBs, shunts the breaks into an HR-mediated repair pathway while preventing 53BP1 binding. We find that the depletion of DDX11 impairs homology-directed repair of DSBs, and this effect is rescued by concomitant *53BP1* silencing, suggesting a role for DDX11 in coupling resection of DSBs with effective HR repair. Two main postresection pathways of RAD51 focus formation have been described, one mediated by BRCA1-PALB2-BRCA2 and the other one by BRCA2-RNF168 (28, 32). Notably, we find that DDX11 is nonredundant with both BRCA1 and BRCA2 branches, as concomitant inactivation of *DDX11* and *BRCA1*/*2* has more severe effects than concomitant silencing of *BRCA1* and *BRCA2* in DNA repair. Mechanistically, we show that DDX11 facilitates resection and formation of a ssDNA substrate suitable for RPA binding, later replaced by RAD51 (Fig. 7*C*), therefore explaining its nonoverlapping functions with BRCA1 and BRCA2. We propose that the DDX11 role in facilitating robust RPA and RAD51 focus formation may be manifested by its ability to unwind certain DNA substrates, such as those containing G4 secondary structures on which DDX11 was shown to act (8, 16) and hairpins (Fig. 7*C*).

Altogether, the results indicate that DDX11 targeting is useful in several cancers by increasing their sensitization to replication stress-induced chemotherapy. DDX11 loss greatly sensitizes both *BRCA1*- and *BRCA2*-deficient cancer cells to chemotherapeutic drugs, generating more DNA damage and genomic instability. Moreover, both *BRCA1*- and *BRCA2*-mutated tumors that acquired resistance via inactivation of 53BP1 and shieldin components or *BRCA2* functional restoration are sensitized by DDX11 loss. We propose that DDX11 provides a mechanism of replication stress tolerance, which sustains survival of cancers, including BRCA1- and BRCA2-deficient cells, and can be exploited therapeutically through the development of specific inhibitors of DDX11 helicase activity.

## Materials and Methods

### Cell Lines and Establishment of Stable Cell Lines.

HeLa (ATCCCCL-2), U2OS (ATCCHTB-96), PEO1, PEO-1 C4-2, Capan-1, and Capan-1 C2-6 [gifts from the Taniguchi laboratory (33, 34)] were cultured in Dulbecco’s Modified Eagle Medium (DMEM) high glucose supplemented with 10% fetal bovine serum (FBS), L-glutamine, and penicillin and streptomycin. The U2OS DIvA (AID-AsiSI-ER-U2OS) cell line from the Legube laboratory (35), was cultured in DMEM high glucose containing G418 (800 μg/mL). The UWB1.289 + BRCA1 (ATCCCRL-2945) cell line was grown in complete 50% Roswell Park Memorial Institute 1640, and 50% mammary epithelial cell growth medium containing G418 (400 μg/mL) was used for the maintenance. All cell lines were tested for mycoplasma contamination and maintained at 37 °C with 5% CO_2_.

For the generation of stable *DDX11* KO cell lines expressing different variants, U2OS *DDX11* KO cells were transfected with plasmids as follows: pCDNA3.1(+) EV, DDX11 WT, DDX11 K50R, DDX11 R263Q, and DDX11 KAE variants. The transfections were performed using Lipofectamine 2000 (Thermo Fisher Scientific). The transfected cells were selected with G418 (1 mg/mL) and cultured in the presence of lower concentration of G418 (500 μg/mL). The expression level of DDX11 variants were analyzed by Western blot.

### siRNA Transfections.

For siRNA transfections, the cells were transfected with 20 to 30 nM of si*DDX11* of Dharmacon ON-TARGETplus siRNA (L-011843-00-0020), ON-TARGETplus Nontargeting Pool (D-001810-10-05), si*53BP1* (SASI_Hs_00024578), si*REV7* (SASI_Hs02_00329127), si*C20orf196* (SASI_ Hs01_ 00102807), si*FAM35A* (SASI_Hs02_00352632), si*BRCA1* (L-003461-00-0005), and si*BRCA2* (L-003462-00-0005) using Lipofectamine RNAiMAX (Invitrogen) according to the manufacturer’s protocol. The depletion was analyzed by immunoblot 48 h post-transfection.

### Generation of *DDX11* KO Cell Lines by CRISPR/Cas9.

To generate HeLa and U2OS *DDX11* KO cell lines, cells were transfected with SpCas9 expressing construct PX459 Addgene (#62988) using Lipofectamine 2000 followed by transfection of Alt-R control guide RNA and Alt-R synthetic paired guide RNAs (Integrated DNA Technologies) that target DDX11 exon 7 (GAG​GTG​AAG​AAG​AGC​CCC​TT) and exon 9 (GGG​CTG​CAG​GGA​TGG​CAA​GG) and then expanded for clonal populations in 96-well plate. The clonal populations were screened by genotyping and the KO cells were confirmed by Western blot and Sanger sequencing. For U2OS *DDX11* KO cells, the *DDX11* genomic loci were PCR amplified by high fidelity Q5 DNA polymerase (New England Biolabs), and the amplified PCR product was cloned in a Zero Blunt TOPO PCR Cloning Kit (Invitrogen). At least 10 to 15 colonies were sequenced to identify frameshifts and deletions. The guide RNAs were designed by using CRISPRscan (https://www.crisprscan.org/) and the CRISPOR online tool (crispor.tefor.net/).

### Colony Formation and Cell Viability Assays.

For colony formation assays, the cells were treated with cisplatin at the indicated concentrations for 1 h and washed thrice with 1× phosphate-buffered saline (PBS) before detaching the cells with trypsin. Around 400 to 500 cells were seeded in 10 cm dishes and allowed to grow for 10 to 15 d. For PARPi and ATRi sensitivity, ∼100 cells were seeded in 10 cm dishes and incubated overnight to allow adherence to the plates. The following day, the cells were treated with olaparib at the indicated concentrations and grown for 10 to 15 d to form individual colonies. The cells were stained with 0.5% crystal violet containing 20% methanol for 30 min at room temperature, and plates were washed with deionized water, and colonies were counted manually. The plating efficiency and surviving fraction was determined by normalizing with untreated cells.

For cell viability assays, 500 to 1,500 cells/well were seeded in 96-well plates and then allowed to adhere to the plates. The drug treatments were chronically given with indicated concentrations of Pyridostatin, Telomestatin, mitomycin C, and olaparib for 3 to 5 d. Cell viability was determined using the 0.5% crystal violet staining containing 20% methanol and normalization with the untreated cells. For siRNA-mediated cell viability assays, cells were incubated in the presence of drugs, and the viability was calculated after 3 to 5 d of incubation. The corresponding Western blots were performed after 48 h of siRNA transfection.

### Western Blot Analysis.

Cell extracts were prepared using radioimmunoprecipitation assay or lysis buffer (50 mM Tris, 250 mM NaCl, 1% Igepal, 0.1/SDS, 5 mM EDTA, 10 mM Na2P2O7, 10 mM NaF) supplemented with Protease Inhibitor Cocktail (Roche) and PhosSTOP, resolved by BioRAD sodium dodecyl sulfate–polyacrylamide gel electrophoresis gels, and transferred to a nitrocellulose membrane followed by incubation with indicated primary and secondary antibodies. All Western blots were performed using at least two independent biological replicates.

### Antibodies for Western Blots and Immunofluorescence.

As primary antibodies, anti-DDX11 (Santa Cruz #sc-271711) (1:1,000), anti-BRCA1 (Santa Cruz #sc-6954) (1:1,000), anti-BRCA2 (ab123491) (1: 1000), anti-Chk2 (Santa Cruz #sc-17747) (1:1,000), anti-pChk2 (pT68) (cell signaling technology #2661) (1:1,000), anti-DNA PKcs (Epitomics #1579–1) (1:1,000), anti-DNA PKcs (pS2056) (Epitomics #3892–1) (1:1,000), anti–γ-H2AX (Millipore #05–636) (1:500), anti-53BP1 (Novus Biologicals #NB100-304) (1:1,000), anti-MAD2B (REV7) (BD Biosciences 612266) (1:1,000), anti-RAD51 (Santa Cruz #sc-17747) (1:50), anti-RPA2 (RPA32) (Thermo Fisher #MA1-26418), and α-tubulin (Santa Cruz #8035) (1:5,000) were used. As secondary antibodies, anti-mouse HRP-linked (1:5,000 cell signaling technology), anti-rabbit HRP-linked (1:5,000 cell signaling technology), and Alexa Fluor 488 anti-mouse (immunofluorescence 1:400) Invitrogen, Alexa Fluor Cy3-conjugated anti-rabbit (immunofluorescence 1:400) Invitrogen were used.

### Chemicals.

The following chemicals were used: olaparib (Selleckchem #S1060), cisplatin (Sigma-Aldrich #479306), telomestatin (Chemexpress 265114–54-3), pyridostatin (Merck #SMLO678), AZD6738 (Selleckchem #S7693), VE-821 (Selleckchem #S8007), and tamoxifen (4OHT) (MedChemExpress #HY-13757A-1g).

### Immunofluorescence.

For 53BP1 and γ−H2AX foci, U2OS cells were grown in coverslips and treated with cisplatin (2.5 μM) for 1 h and then washed thrice with 1× PBS followed by fixation with 4% formaldehyde in 1× PBS for 15 to 20 min with samples taken at the indicated time points. The coverslips were washed thrice with 1× PBS followed by permeabilization with 0.3% Triton X-100 in 1× PBS for 5 min at room temperature and washed thrice with 1× PBS and then blocked with 10% horse serum after the washes. RAD51 and RPA32 immunofluorescence was performed as previously described in ref. 22. Coverslips were incubated with indicated primary antibodies (53BP1 [1:1,000], RAD51 [1:50], and γ−H2AX [1:500]) for 2 h in room temperature and then washed thrice with 1× PBS before incubation with secondary antibodies (Alexa Fluor 488 and Cy3 [1:400]) for 1 h. After the incubation with secondary antibodies, the coverslips were washed with 1× PBS thrice and stained with DAPI for 20 min, and then images were taken with a confocal microscope (Leica TCS SP2 AOBS inverted). The 53BP1, RAD51, and γ−H2AX foci were analyzed by Fiji and cell profiler software. For foci analysis, at least 100 nuclei were analyzed for each time point except for Fig. 7*A* in which at least 75 nuclei were analyzed.

For analysis of micronuclei/mitotic catastrophe, the cells were grown on coverslips. The cells were treated with cisplatin (2.5 μM) for 1 h and washed thrice with 1× PBS. For PARPi, cells were grown in the presence of olaparib (1μM) followed by the fixation of cells at a final concentration of 4% paraformaldehyde for 10 to 15 min at the indicated time points, and then cells were washed with 1× PBS thrice. Furthermore, the cells were stained with DAPI and scored for the indicated phenotypes. For micronuclei/mitotic catastrophe, at least 100 nuclei were counted. All immunofluorescence experiments were performed in at least two independent biological replicates.

### DR-GFP Assay.

U2OS TRI DR-GFP cells (27) were transfected with the indicated siRNAs (20 to 30 nM) using Lipofectamine RNAiMAX (Invitrogen), and I-SceI endonuclease was induced using doxycycline (5 μg/mL) addition after 48 h of post siRNA transfection. The cells were fixed with a formaldehyde solution after 72 h of doxycycline addition. All samples were processed according as in ref. 37 and analyzed by fluorescence-activated cell sorting.

### Resection Assay.

U2OS DIvA (AID-AsiSI-ER-U2OS) cells were transfected with the indicated siRNA using Lipofectamine RNAiMAX. After 48 h of post-transfection, cells were treated with 4OHT (300 nM) for 4 h to induce AsiSI-dependent DSB induction. The collected cells were lysed, and DNA was extracted using a DNAeasy kit (Qiagen). Briefly, in total, 500 to 1,000 ng of isolated genomic DNA was digested using a Ban1 restriction enzyme (New England Biolabs) at 37 °C overnight. The restriction enzyme cuts genomic DNA ∼200 bp from the DSB-KDELR3 and ∼740 bp for DSB-ASXL1. RNase treatment was given to digested and undigested genomic DNA samples and incubated at 65 °C for 20 min for the heat inactivation of enzymes. Genomic DNA samples were analyzed by real-time PCR using the following primers:

DSB-KDELR3_200 FW: ACC​ATG​AAC​GTG​TTC​CGA​AT,
DSB-KDELR3_200_REV: GAG​CTC​CGC​AAA​GTT​TCA​AG,
DSB-ASXL1_740 FW: GTC​CCC​TCC​CCC​ACT​ATT​T,
DSB-ASXL1_740_REV: ACG​CAC​CTG​GTT​TAG​ATT​GG,
DSB-KDELR3_20kb_FW: CAC​TCA​TCC​TGA​TAC​ATC​AG, and
DSB-KDELR3_20kb_REV: TAC​AGT​ACT​AAT​TGG​GAG​GC.

ssDNA percentage was calculated as described in ref. 38). The DNA amount was normalized for each sample using a control region at 20 kb away from the AsiSI cut site at the KDELR3 locus.

### Synthetic Lethality Chemical Drug Screens.

For high content drug screens, the drug plates were prepared with various concentrations in 384-well plates. HeLa Ctrl and *DDX11* KO cells were seeded on a 384-well plate using a Multidrop 384 dispenser Titertek (Thermo LabSystems, Inc.) and incubated for 72 h in the presence of drugs. Cell viability was analyzed by using CellTiter-Glo, and readings were taken in 384-well StorPlate-384V (#6008598, PerkinElmer, Inc.). For Fig. 1*D*, the image was prepared using the BioRender online software.

### Statistical Methods.

Statistical analysis was performed using GraphPad prism software. Statistical differences for experiments are mentioned in figure legends. Prism software versions 8 and 9 were used to prepare graphs and analyze statistical significance.

### Kaplan–Meyer Curves.

We used the Kaplan–Meier Plotter web tool (https://kmplot.com/analysis/) to compare the overall survival of patients divided in two groups by *DDX11* median expression in ovarian and lung cancer data.

## Supplementary Material

Supplementary File

Supplementary File

## Data Availability

Source data have been deposited in Mendeley (DOI: 10.17632/tz4z2syb2r.1). All other study data are included in the article and/or supporting information.
