# Supplementary material for: DDX11 loss causes replication stress and pharmacologically exploitable DNA repair defects
Source: Proc Natl Acad Sci U S A. 2021 Apr 20;118(17):e2024258118. doi: 10.1073/pnas.2024258118 (PMC8092582; doi:10.1073/pnas.2024258118)
Supplement: Supplementary File [file pnas.2024258118.sapp.pdf]

Supplementary Information Appendix

Targeting DDX11 in cancers causes replication stress and pharmacologically exploitable DNA repair defects

Nanda Kumar Jegadesan[1] and Dana Branzei[1, 2, *]

[1] IFOM, the FIRC Institute of Molecular Oncology, Via Adamello 16, 20139, Milan, Italy

[2] Istituto di Genetica Molecolare, Consiglio Nazionale delle Ricerche (IGM-CNR), Via Abbiategrasso 207, 27100, Pavia, Italy

[*] Corresponding author

E-mail: dana.branzei@ifom.eu (D.B.)

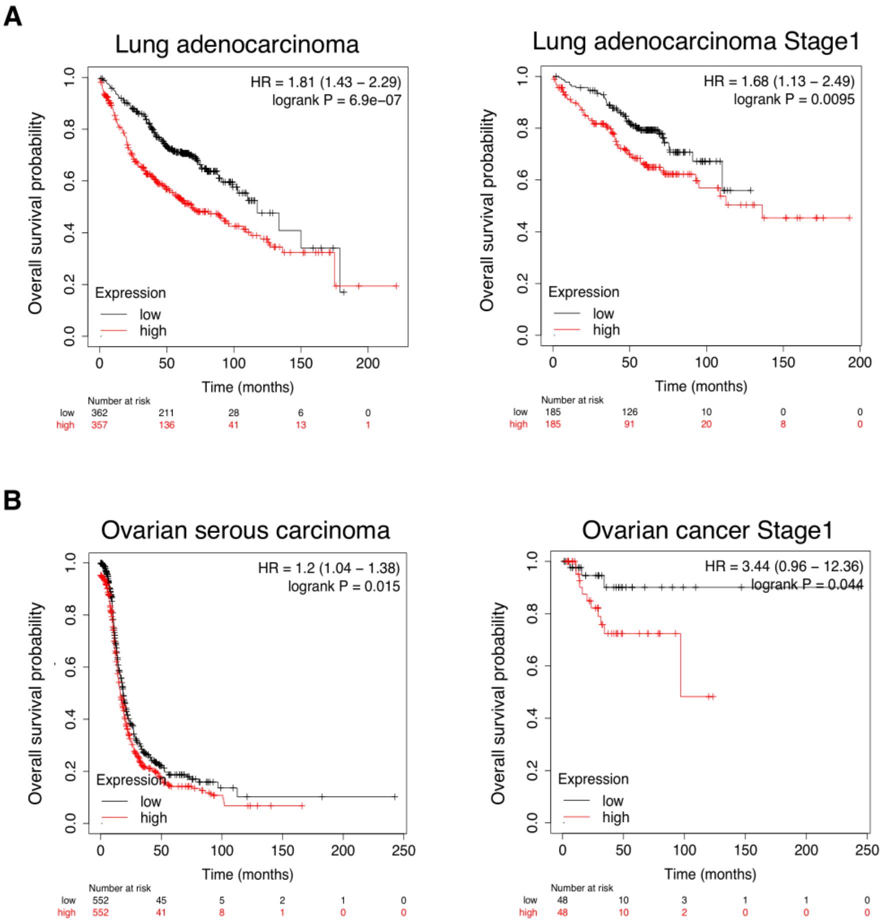

Fig. S1. Kaplan–Meier survival probability plots stratified by DDX11 expression levels

A and B, Kaplan–Meier overall survival plot of patients with lung adenocarcinoma and ovarian serous carcinoma, stratified by DDX11 median expression level. Patients sample numbers are indicated below the Kaplan-Meier plots.

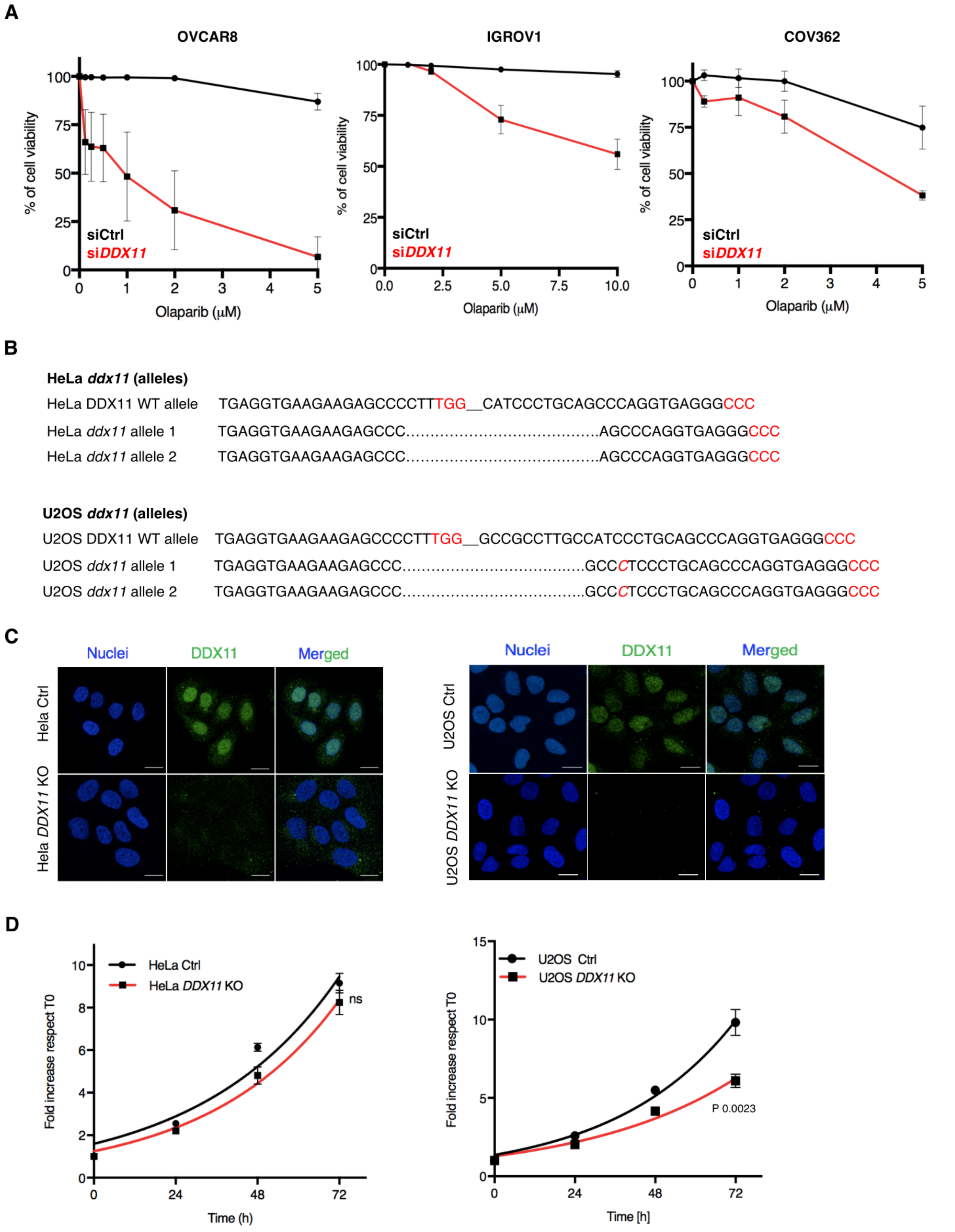

Fig. S2 Loss of DDX11 sensitizes ovarian cancer cell lines and establishment of *DDX11* knockout

A, Sensitivity assay of different ovarian cancer cell lines transfected with siCtrl and si*DDX11* upon Olaparib treatment with the indicated drug concentrations (n=3 for OVCAR8 and COV362 and n=2

for IGROV1). Cell viability was determined using crystal violet staining after 5 days of drug treatment. Error bars show average ± SEM. **B,** Sangers sequencing of *DDX11* genomic loci of HeLa and U2OS Ctrl and *DDX11* KO cells respectively. **C,** Immunofluorescence analysis of DDX11 in HeLa and U2OS Ctrl and *DDX11* KO cells respectively. **D,** Cellular proliferation assay in HeLa and U2OS Ctrl and *DDX11* KO, respectively, using CellTiter-Glo at the indicated time points (n=3). Error bars show average ± SEM.

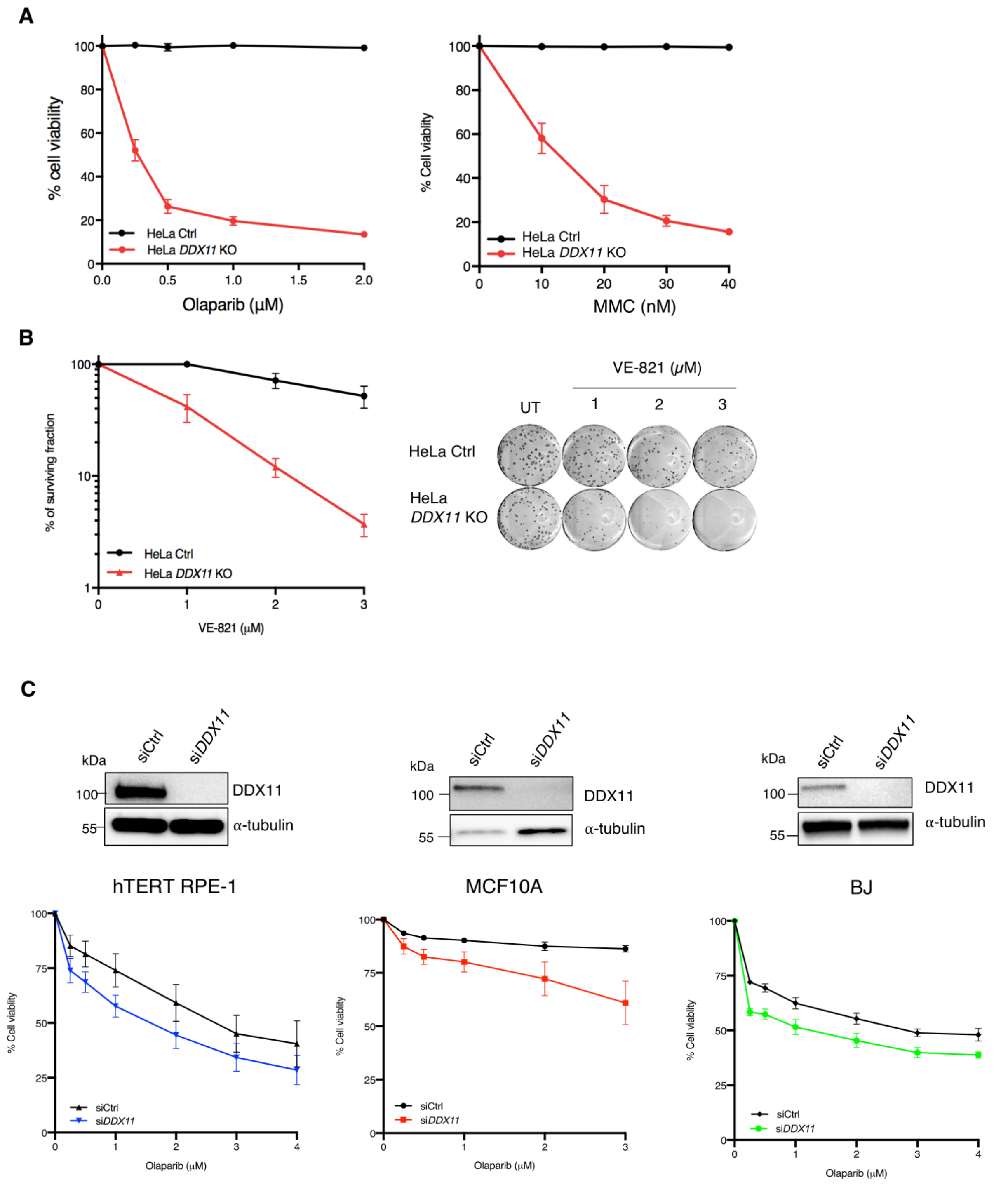

Fig. S3 Validation of synthetic lethal drug combinations with *DDX11* knockout

A, Cell viability assay of HeLa Ctrl and *DDX11* KO cells treated with Olaparib and Mitomycin-C (MMC) with the indicated concentrations (n=3). Cell viability was determined using crystal violet after 5 days of incubation. Error bars show average ± SEM. **B,** Colony formation assay of HeLa Ctrl and *DDX11* KO cells treated with ATR inhibitor VE-821 (n=3) with the indicated drug concentrations. Colonies were stained with crystal violet after 10 to 15 days of incubation. Error bars show average ± SEM. **C,** Cell viability assay of hTERT RPE-1, MCF10A and BJ cells transfected

with siCtrl and si*DDX11*. Cells were treated with Olaparib with the indicated concentrations (n=3). Cell viability was determined using crystal violet after 5 days of incubation. Error bars show average ± SEM.

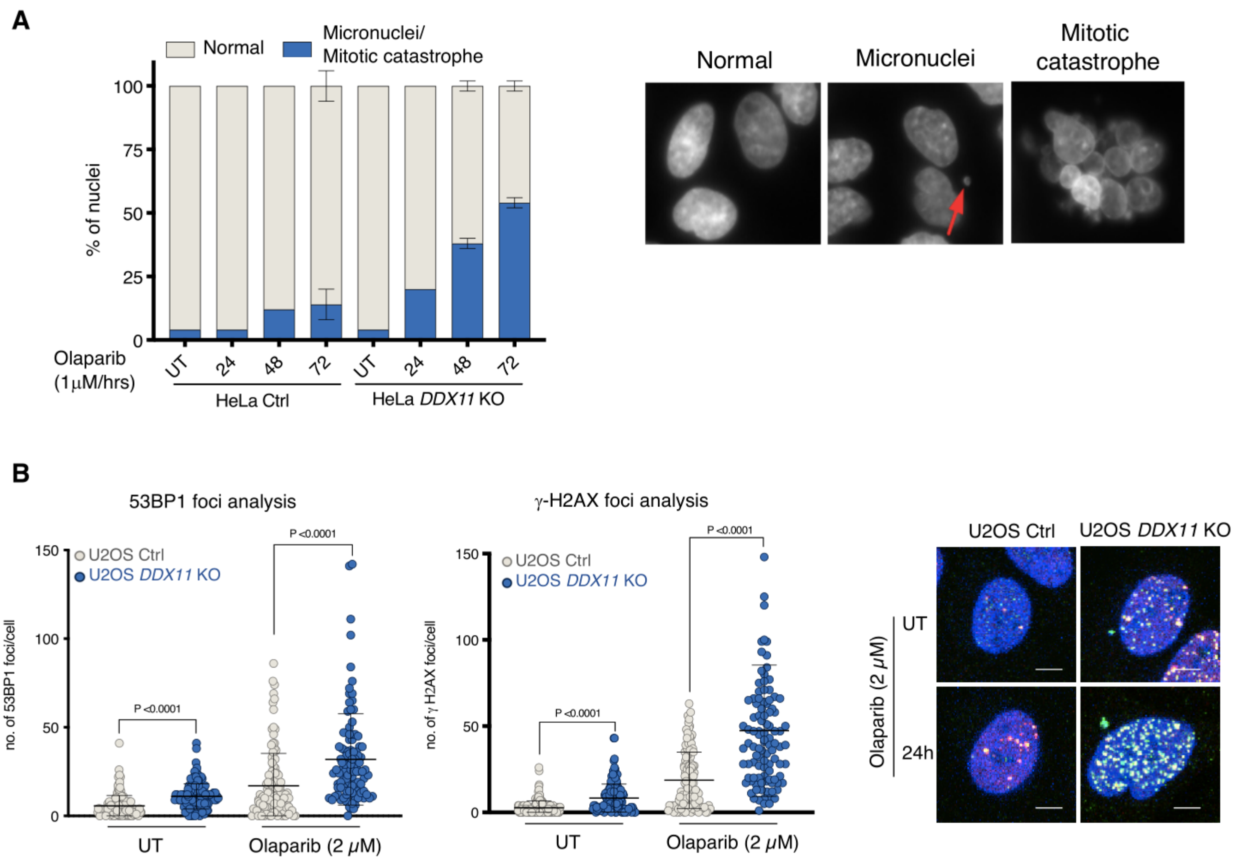

Fig. S4 DDX11 loss associates with micronucleation and DNA damage accumulation

A, Quantification of micronuclei and mitotic catastrophes in HeLa Ctrl and *DDX11* KO cells in untreated conditions and upon Olaparib (1 µM) drug treatment for the indicated time points. Error bar shows average ± SEM. **B,** Representative micrographs of 53BP1 and γ-H2AX focus formation in U2OS Ctrl and *DDX11* KO cells upon Olaparib (2 µM) drug treatment for 24 hours (scale bar, 10 µM). Quantification of foci is shown (n=2). Statistical analysis was performed using Students *t*-test. Error bar shows average ± SD.

A

B

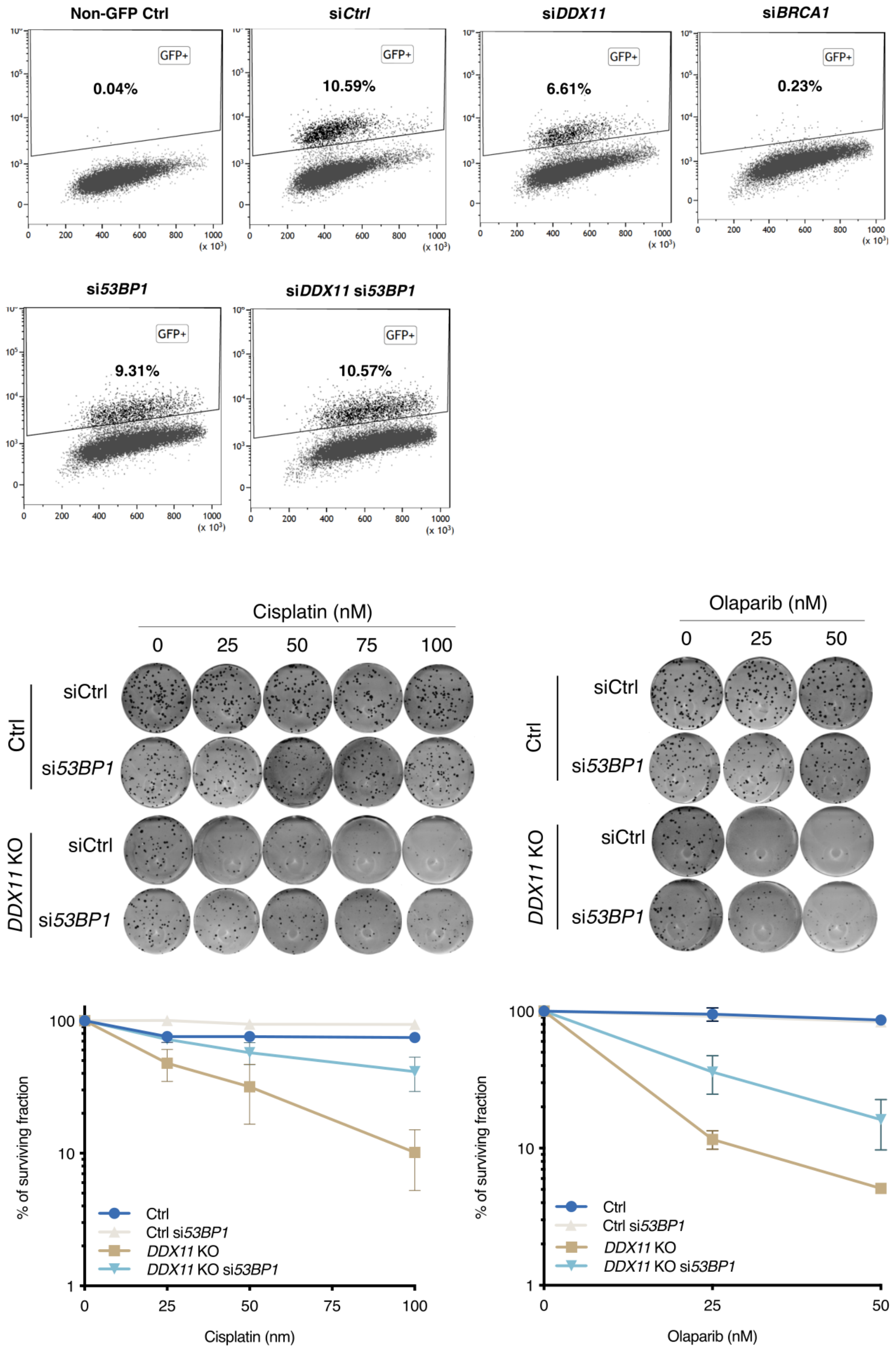

Non-GFP Ctrl
0.04%
siCtrl
10.59%
siDDX11
6.61%
siBRCA1
0.23%
si53BP1
9.31%
siDDX11 si53BP1
10.57%
GFP+
Cisplatin (nM)
0
25
50
75
100
Olaparib (nM)
Ctrl
DDX11 KO
siCtrl
si53BP1
% of surviving fraction
Cisplatin (nm)
Olaparib (nM)
Ctrl
Ctrl si53BP1
DDX11 KO
DDX11 KO si53BP1

Fig. S5 DDX11 promotes homology-directed repair of DSBs downstream of 53BP1

A, Representation of flow cytometry scatter plot analysis of U2OS TRI DR-GFP cells transfected with indicated siRNAs for Fig. 4B. The analysis was performed 72 hours after I-SceI induction by doxycycline (n=3). Error bar shows average ± SEM. **B**, Colony formation assay of HeLa Ctrl and *DDX11* KO cells transfected with the indicated siRNAs. Cell were chronically treated with Cisplatin (n=3) and Olaparib (n=2) and colonies were stained with crystal violet after 10 to 15 days of incubation. Error bar shows average ± SEM.

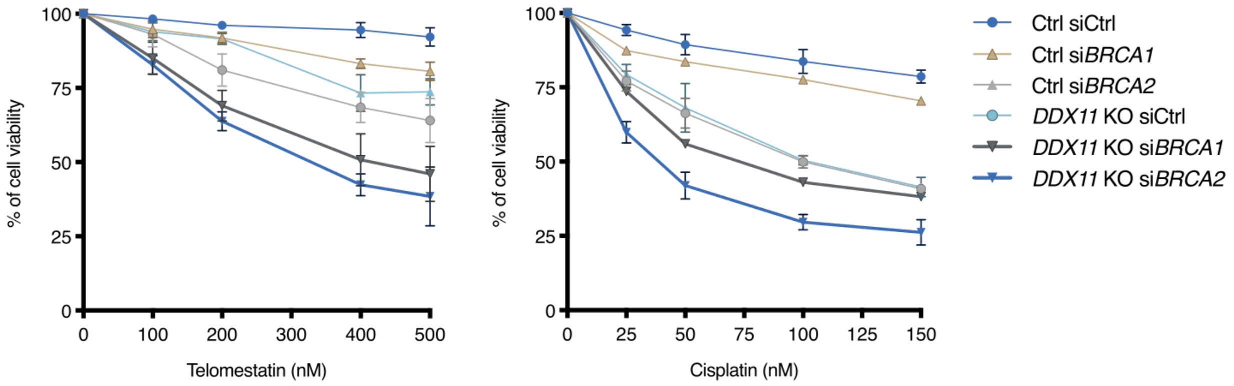

Fig. S6 DDX11 has complementary functions to both BRCA1 and BRCA2 upon DNA damage

HeLa Ctrl and *DDX11* KO cells were transfected with indicated siRNAs. Cell viability was determined using crystal violet staining after 5-6 days of incubation with the indicated concentrations of Telomestatin and Cisplatin, n≥3. Error bars show average ± SEM.

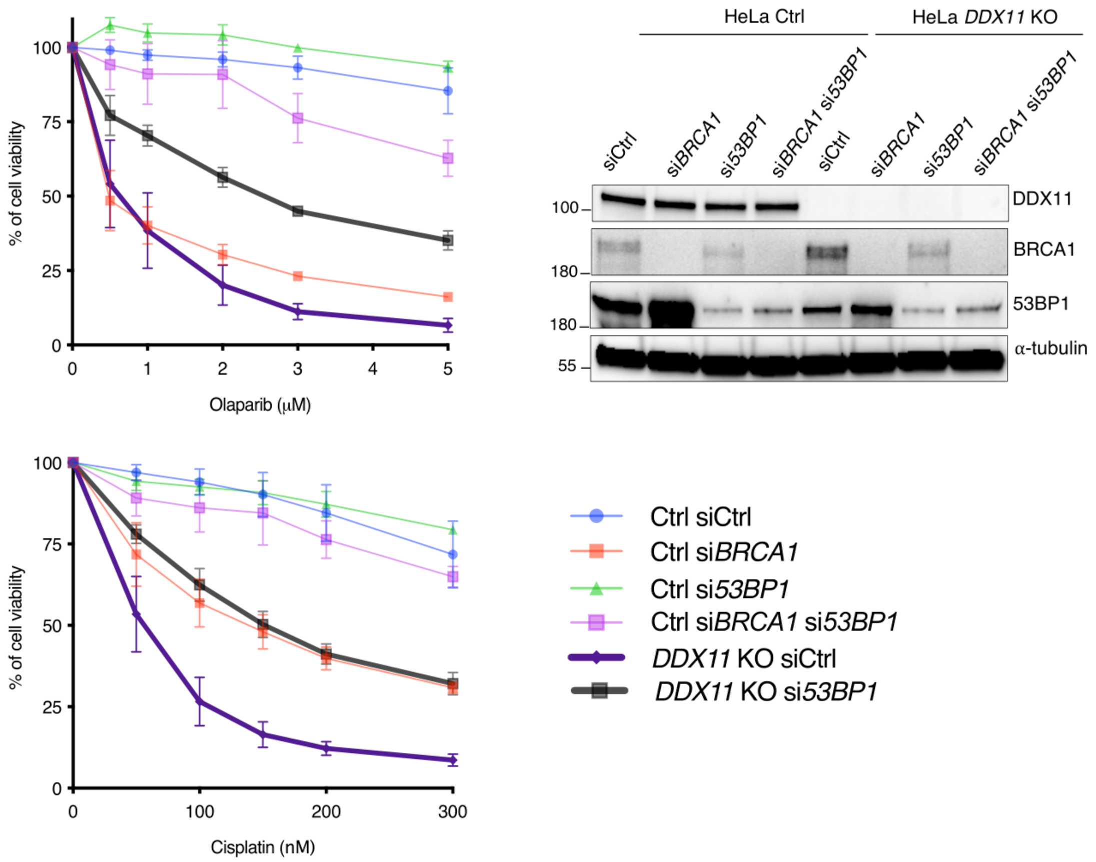

Fig. S7. *DDX11* KO sensitivity is suppressed by 53BP1 depletion. HeLa Ctrl and *DDX11* KO cells were transfected with indicated siRNAs. Cell viability was determined using crystal violet staining after 5-6 days of incubation with the indicated concentrations of Olaparib and Cisplatin, n=3. Error bars show average ± SEM. Representative Western blot showing the depletion of indicated proteins using siRNAs is shown.

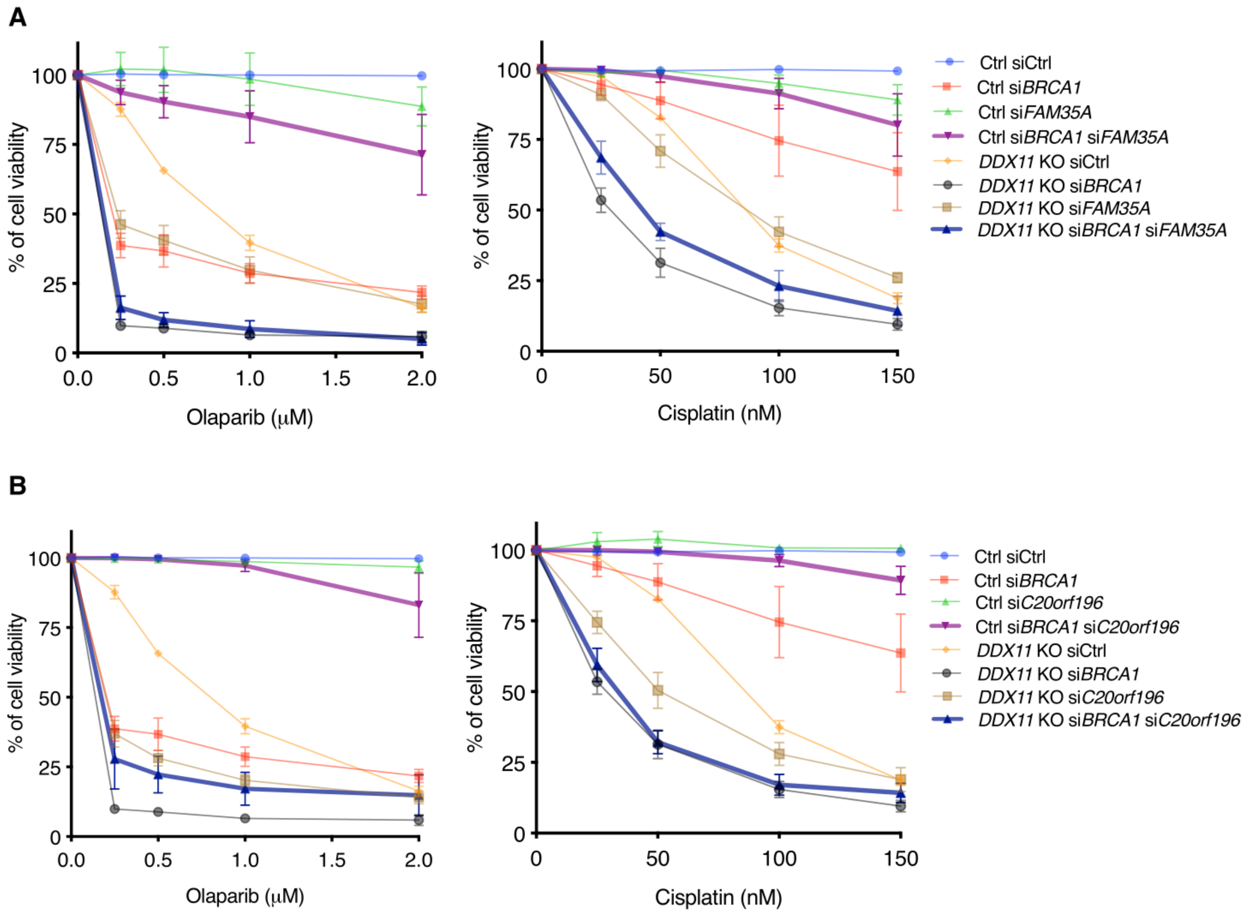

Fig. S8 DDX11 is required for viability in *BRCA1* Shieldin depleted cells exposed to chemotherapeutic drugs. A and B, Cell viability assay of HeLa Ctrl and *DDX11* KO cells transfected with indicated siRNAs. Cells were treated with Olaparib and Cisplatin with the indicated drug concentrations for 6 days (n=3). Cell viability was determined by using crystal violet staining. Error bars show average ± SEM.

Supplementary Table 1. Summary of drug screen results for HeLa *DDX11* knockout

Name	Target	HeLa DDX11 KO
(+) JQ-1	BRD4	S
Abitrexate (Methotrexate)	DHFR	-
ABT-263 (Navitoclax)	Bcl-2	-
Adrucil (Fluorouracil)	DNA/RNA Synthesis	-
Alisertib	Aurora A	-
Alvocidib (Flavopiridol)	CDK	-
Aphidicolin	DNA polymerase	-
AT9283	Aurora, Abl, JAK2	-
Azacitidine	DNA methyltransferase	-
AZD1152-HQPA (Barasertib)	Aurora B	-
AZD1775	WEE1	-
AZD5363	AKT	S
AZD6738	ATR	S
AZD7762	CHK1/2	S
AZD8186	PI3K	S
AZD8835	PI3K	S
BAY-1895344	ATR	-
Bleomycin sulfate	DNA/RNA Synthesis	S
Bortezomib (Velcade)	Proteasome	-
Buparlisib (BKM-120)	PI3K	-
Carboplatin	DNA/RNA Synthesis	S
Carfilzomib	Proteasome	S
Carmofur	DNA/RNA Synthesis	-
Chlorambucil	DNA/RNA Synthesis	-
Cisplatin	DNA/RNA Synthesis	S
Cladribine	DNA/RNA Synthesis	-
Clofarabine	DNA/RNA Synthesis	-
Cytarabine	DNA/RNA Synthesis	S
Danusertib (PHA-739358)	Aurora kinase	-
Decitabine	DNA/RNA Synthesis	-
Dinaciclib	CDK	-
Doxorubicin (Adriamycin)	Topoisomerase	S
Epirubicin Hydrochloride	Topoisomerase	S
Etoposide (VP-16)	Topoisomerase	S
Floxuridine	DNA/RNA Synthesis	-
Fludarabine Phosphate (Fludara)	DNA/RNA Synthesis	-
Ftorafur (Tegafur)	DNA/RNA Synthesis	-
Gemcitabine HCl (Gemzar)	DNA/RNA Synthesis	-
Hesperadin	Aurora B	M
Iniparib (BSI-201)	PARP1	-
KU-55933	ATM, DNA-PK, PI3K	S

Mitoxantrone Hydrochloride	Topoisomerase	S
NU7441	DNA-PK	-
Nutlin-3	p53/MDM2	-
Obatoclax mesylate	Bcl-2	-
Olaparib	PARP1/2	S
Omipalisib (GSK2126458)	PI3K	-
Paclitaxel (Taxol)	Microtubule Associated	-
Palbociclib	CDK4/6	-
Pemetrexed	DHFR, DNA/RNA Synthesis	-
Ralimetinib (LY2228820)	p38 MAPK	S
Rapamycin (Sirolimus)	mTOR	S
RITA	p53/MDM2	M
Seliciclib (Roscovitine)	CDK1/2/5	-
Selumetinib (AZD6244)	MEK1/2	-
Thioguanine	DNA methyltransferase	R
Topotecan HCl	Topoisomerase	-
Tozasertib (VX-680)	Aurora A	-
Trabectedin (Yondelis)	DNA synthesis	-
Trametinib	MEK1/2	S
Vinblastine	Microtubule Associated	-
Vistusertib (AZD2014)	mTOR	S
Vorinostat (SAHA)	HDAC	-
YM155 (Sepantronium bromide)	Survivin	-

Abbreviation:
Sensitization (S), Resistance (R), Moderate (M)

Data Set Table 2 available as Excel File.
